# Deleterious variants in *DCHS1* are prevalent in sporadic cases of mitral valve prolapse

**DOI:** 10.1002/mgg3.347

**Published:** 2017-12-10

**Authors:** Alisson Clemenceau, Jean‐Christophe Bérubé, Paméla Bélanger, Nathalie Gaudreault, Maxime Lamontagne, Oumhani Toubal, Marie‐Annick Clavel, Romain Capoulade, Patrick Mathieu, Philippe Pibarot, Yohan Bosse

**Affiliations:** ^1^ Institut Universitaire de Cardiologie et de Pneumologie de Québec Quebec QC Canada; ^2^ Department of Molecular Medicine Laval University Quebec QC Canada

**Keywords:** *DCHS1*, deleterious variants, genetics, mitral valve prolapse, sequencing

## Abstract

**Background:**

A recent study identified *DCHS1* as a causal gene for mitral valve prolapse. The goal of this study is to investigate the presence and frequency of known and novel variants in this gene in 100 asymptomatic patients with moderate to severe organic mitral regurgitation.

**Methods:**

DNA sequencing assays were developed for two previously identified functional missense variants, namely p.R2330C and p.R2513H, and all 21 exons of *DCHS1*. Pathogenicity of variants was evaluated in silico.

**Results:**

p.R2330C and p.R2513H were not identified in this cohort. Sequencing all coding regions revealed eight missense variants including six considered deleterious. This includes one novel variant (p.A2464P) and two rare variants (p.R2770Q and p.R2462Q). These variants are predicted to be deleterious with combined annotation‐dependent depletion (CADD) scores greater than 25, which are in the same range as p.R2330C (CADD = 28.0) and p.R2513H (CADD = 24.3). More globally, 24 of 100 cases were carriers of at least one in silico‐predicted deleterious missense variant in *DCHS1*, suggesting that this single gene may account for a substantial portion of cases.

**Conclusion:**

This study reveals an important contribution of germline variants in *DCHS1* in unrelated patients with mitral valve prolapse and supports genetic testing of this gene to screen individuals at risk.

## 1 INTRODUCTION

Targeted sequencing of a locus linked to mitral valve prolapse (MVP) on chromosome 11p15.4 in four affected members of an extended family revealed protein‐altering variants in the *DCHS1* gene (Durst et al., 2015) (OMIM #603057). A loss‐of‐function variant, labeled p.R2513H, in exon 21 segregated with the disease in this large pedigree. In two additional families, a second protein damaging variant, p.R2330C, in *DCHS1* segregated with mitral valve prolapse. Mitral valve interstitial cells transfected with cDNA constructs demonstrated reduced protein stability of mutants (p.R2513H and p.R2330C) compared to wild‐type alleles. In the same study, Dchs1‐deficient mice (Dchs1^+/−^) exhibited mitral valve prolapse confirming that loss of function of this gene results in disease.

Mitral valve prolapse is a common disease affecting approximately 2.4% of the population (Freed et al., 1999). The contribution of rare *DCHS1* genetic variants to sporadic form of MVP remains to be determined. The goal of this study is to investigate the presence of p.R2513H and p.R2330C in a series of unrelated French Canadian patients with MVP and also to comprehensively screen the coding regions of *DCHS1* to find other potentially deleterious variants.

## 2 MATERIALS AND METHODS

### 2.1 Ethical compliance

All patients provided written informed consent and the study was approved by the ethics committee of the *Institut universitaire de cardiologie et de pneumologie de Québec* (IUCPQ) (20758 and 20341).

### 2.2 The Quebec City MVP cohort

One hundred asymptomatic patients with MVP and at least moderate organic mitral regurgitation (MR) (defined as an effective regurgitant orifice area ≥20 mm² and/or a regurgitant volume ≥30 ml) (Enriquez‐Sarano, Akins, & Vahanian, 2009), preserved left ventricular (LV) ejection fraction (>60%), and normal LV end‐systolic diameter (<45 mm) were prospectively recruited at the IUCPQ, Quebec, Canada. Patients with the following criteria were excluded: (1) MR due to ischemic heart disease or cardiomyopathy; (2) >mild mitral stenosis, aortic regurgitation, aortic stenosis, or pulmonary stenosis; (3) previous valve operation; (4) history of myocardial infarction or angiographically documented coronary artery stenosis; (5) congenital or pericardial heart disease; and (6) endocarditis. All patients underwent an electrocardiogram and Doppler echocardiography examinations. The quantification of MR was assessed by proximal isovelocity surface area (PISA) method and by two volumetric quantitative Doppler methods based on the principle of the continuity equation (Enriquez‐Sarano, Seward, Bailey, & Tajik, 1994; Enriquez‐Sarano et al., 2005; Magne et al., 2007, 2014). No patients were carriers of mutations in the *FLNA* gene (G288R, P637Q, V711D, and the 1,944‐bp deletion) known to cause isolated nonsyndromic mitral valve prolapse (Kyndt et al., 2007).

### 2.3 DNA sequencing of *DCHS1*

DNA was extracted from 200 μl of frozen whole blood using QIAamp^®^ DNA Blood Mini kit (Qiagen). The DNA quality and concentration was assessed by the UV absorbance ratio 260/280 nm and UV absorbance 260 nm, respectively. DNA fragments containing the p.R2330C and p.R2513H variants were amplified and read by Sanger sequencing in 100 patients. The DNA sequences of all coding and untranslated regions (i.e., exons 1–21) of the *DCHS1* gene were then obtained by Sanger sequencing in a randomly selected subset of 12 patients. Coding regions identified with deleterious variants in this subset of patients were sequenced in the remaining 88 samples. Primer sequences to evaluate the selected regions of *DCHS1* are provided in Table S1. PCR was performed in a final volume of 25 μl containing 100 ng of genomic DNA, 1 U of HotStar Taq DNA polymerase (Qiagen), PCR buffer 1×, Q‐Solution 1×, 160 μmol/L of each dNTP, and 0.2 μmol/L of each primer. Exons 1 and 10 required the addition of 160 μmol/L of 7‐deaza‐dGTP and 5% DMSO, respectively. The PCRs were carried out on either GeneAmp^®^ PCR system 9700 or Veriti Thermal Cycler (Applied Biosystems^®^). Cycling conditions were adapted to the size and GC content of each amplicon (Table S1). A modified touchdown cycling method was used for some coding regions consisting of 11–15 cycles where the annealing temperature was decreased by 0.5°C every cycle from 67 to 58–62°C followed by 20–35 additional cycles with a fixed annealing temperature. Exon 1 was amplified using a slowdown cycling method for GC‐rich region (Bachmann, Siffert, & Frey, 2003). The sequencing reaction was then performed using standard procedures and the product was run on the ABI 3730xl DNA Analyzer (Applied Biosystems^®^). Sequencing files were assembled and analyzed using the EMBL‐EBI Clustal Omega Multiple Alignment Tool (http://www.ebi.ac.uk/Tools/msa/clustalo). Newly identified variants were named based on standard gene mutation nomenclature (den Dunnen et al., 2016) with nucleotide number based on the reference sequence NG_033858.1.

### 2.4 In silico functional analysis of genetic variants

Pathogenicity of genetic variants was evaluated using PolyPhen (Adzhubei et al., 2010), the combined annotation‐dependent depletion (CADD) framework (Kircher et al., 2014), an unsupervised spectral approach (Eigen) (Ionita‐Laza, McCallum, Xu, & Buxbaum, 2016), the CONsensus DELeteriousness score of missense mutations (Condel) (Gonzalez‐Perez & Lopez‐Bigas, 2011), the Mendelian Clinically Applicable Pathogenicity (M‐CAP) score (Jagadeesh et al., 2016), and the Protein Variation Effect Analyzer (PROVEAN) (Choi, Sims, Murphy, Miller, & Chan, 2012). Allele frequencies of identified variants were compared to publically available databases including the 1000 Genomes Project (The 1000 Genomes Project Consortium, 2015), the Exome Aggregation Consortium (ExAC) and the Genome Aggregation Database (gnomAD) (Lek et al., 2016), the Human Longevity Inc (HLI) 10,000 genomes (Telenti et al., 2016), and the variant browser Bravo from the NHLBI's TOPMed program (https://bravo.sph.umich.edu/).

## 3 RESULTS

**Figure 1:**
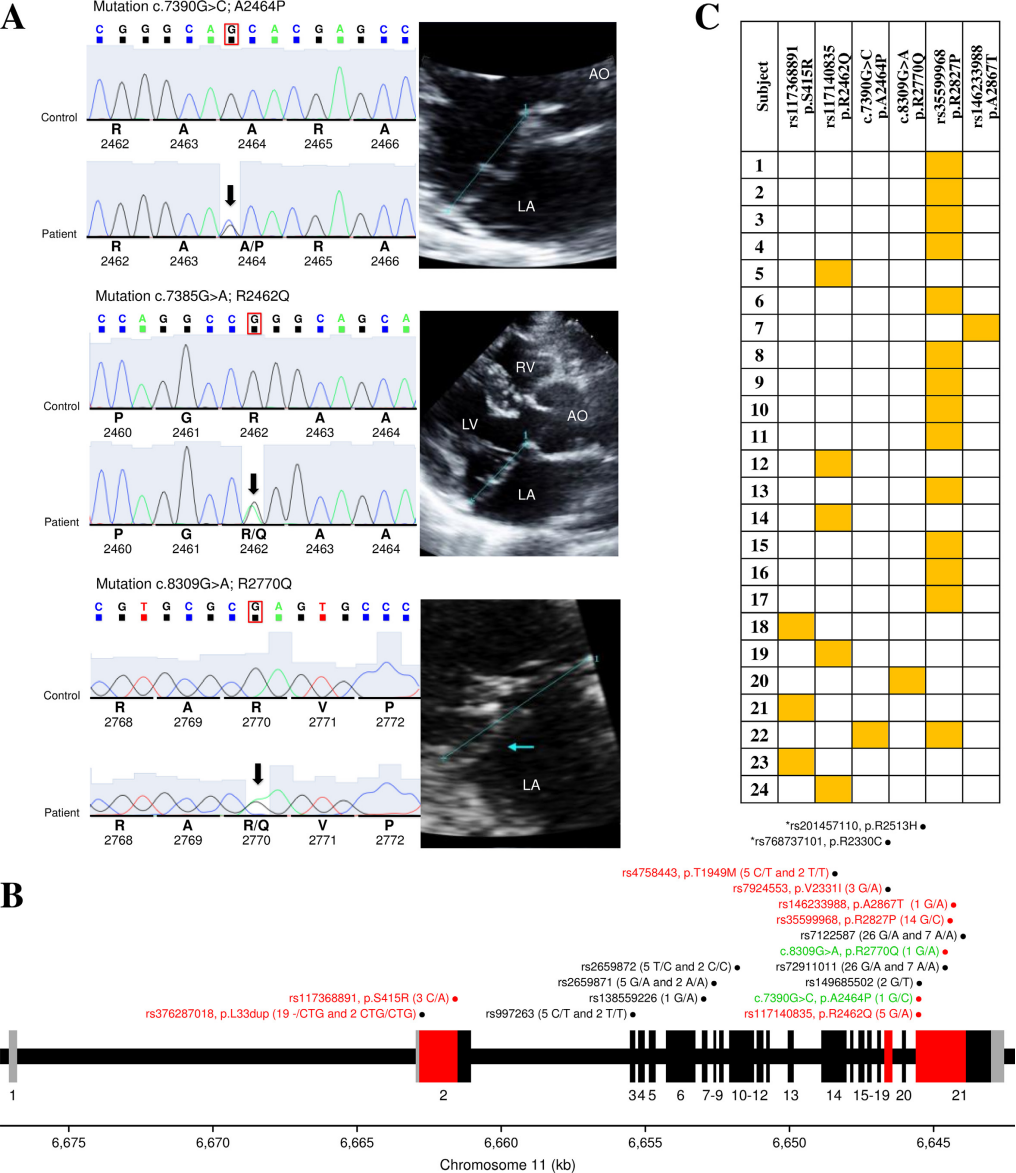
Identification and characterization of genetic variants in the *DCHS1* gene in patients affected by mitral valve prolapse. (a) Sequence chromatograms of the novel (p.A2464P) and rare (p.R2462Q and p.R2770Q) missense in silico‐predicted deleterious variants identified in this study with two‐dimensional echocardiographic long‐axis view of a representative heterozygote patient for each variant. The blue line denotes the mitral annulus. (b) The exon–intron structure of the *DCHS1* gene and the localization of the identified genetic variants. The coding exons are shown in black (or red) and the untranslated regions in gray. The regions of the gene sequenced among the 100 patients are in red. Genetic variants are illustrated with their rs numbers if available with genotyping counts in parentheses for 12 or 100 patients. Red dots illustrate the six in silico‐predicted deleterious variants. The two functional variants identified by Durst et al. (2015) on exons 19 and 21 are illustrated on top in black with an asterisk. Missense and synonymous variants identified in this study are indicated in red and black, respectively. Newly and rare identified variants with no rs number are illustrated in green and named based on standard gene mutation nomenclature (den Dunnen et al., 2016). (c) Summary of patients carrying at least one of six variants identified and considered deleterious in this study. Heterozygote carriers are identified by a yellow box

The clinical characteristics of patients are indicated in Table 1. Two DNA fragments containing the p.R2513H and p.R2330C variants were amplified and sequenced among the 100 patients with MVP. No patient was carrier of these two variants. In exon 19, one missense variant (rs7924553, p.V2331I) was identified in three heterozygote patients. Interestingly, this variant occurs at one amino acid residue next to the loss of function p.R2330C identified by Durst et al. (2015), but was considered benign based on bioinformatics tools to evaluate pathogenicity. In exon 21, one synonymous variant (rs149685502, p.V2470V) and two missenses were identified including p.R2462Q (rs117140835) and p.A2464P found in 5 and 1 heterozygote patients, respectively. p.A2464P has never been observed before and is characterized by a G to C substitution (c.7390G>C) resulting in a proline instead of an alanine at position 2,464 of the protein (Figure 1a). This new variant as well as p.R2462Q are predicted to be protein damaging at the same extent as p.R2513H and p.R2330C (Table 2 and Table S2). Together, by sequencing exon 19 and part of exon 21 of *DCHS1*, we confirmed the absence of p.R2513H and p.R2330C in our population, but revealed two additional in silico‐predicted deleterious variants: p.R2462Q (rs117140835) and p.A2464P.

**Table 1:** Clinical characteristics of patients with mitral valve prolapse

Characteristics	Cases (*n* = 100)	Subset (*n* = 12)
Age (years)	61.1 ± 14.7	58.3 ± 13.1
Gender (% male)	54	58
Diabetes (%)	4	0
Hypertension (%)	35	25
BMI (kg/m^2^)	24.3 ± 3.8	24.2 ± 3.1
Cholesterol (mmol/L)	5.1 ± 1.1 (2)	5.4 ± 1.0
Triglycerides (mmol/L)	1.2 ± 0.7 (2)	1.2 ± 0.4
LDL (mmol/L)	2.8 ± 1.0 (2)	3.1 ± 0.9
HDL (mmol/L)	1.7 ± 0.5 (2)	1.8 ± 0.5
Effective regurgitant orifice area (mm^2^)	29.0 ± 14.5	29.8 ± 17.3
Regurgitant volume (ml)	54.5 ± 28.1	60.0 ± 47.9
Valve leaflet prolapse or flail		
Anterior leaflet prolapse (%)	12	17
Posterior leaflet prolapse (%)	26	25
Bileaflet prolapse (%)	30	25
Posterior leaflet flail (%)	17	25
Valve leaflet remodeling with no prolapse or flail (%)	12	8
Parachute valve^a^ (%)	1	0
Unknown (%)	2	0
Clinical events during follow‐up^b^		
Cardiac arrest (%)	1	0
Heart failure (%)	2	0
Atrial fibrillation (%)	9	8
Ventricular arrhythmia (%)	2	0
None (%)	86	92
Surgery during follow‐up^b^		
Mitral valve repair (%)	33	33
Mitral valve replacement (%)	14	17
None (%)	52	50
Unknown (%)	1	0
Concomitant coronary artery bypass grafting (%)	3	0
Concomitant implantation of a defibrillator (%)	1	0

Continuous variables are *M* ± *SD*. Number of missing values is shown in parenthesis when applicable.

a Congenital abnormality, all chordae tendineae of both leaflets are inserted in a single papillary muscle.

b All patients were asymptomatic and free of previous surgery at baseline. Clinical events and surgeries have occurred during the follow‐up.

**Table 2:** List of genetic variants identified, indexes of pathogenicity, and minor allele frequencies in publically available datasets

# of subject sequenced	Variation	Position		Function	Alleles		Genotypes	mRNA	Protein	Pathogenicity		MAF	
		Exon/intron	B38		Ref	Alt				PolyPhen2	CADD	1,000 All	ExAC adjusted
12	g.6655974_6655975ins (New)	Intergenic	6,655,974	Intergenic	—	G	2 –/G				8.9		
12	rs867488547	E 1	6,655,846	5′ UTR	C	G	2 C/G				7.0	0	
12	rs867009988	E 1	6,655,844	5′ UTR	G	A	6 G/A				9.4	0	
12	g.6655824_6655825ins	E 1	6,655,824	5′ UTR	—	AGGGG	6 –/AGGGG				9.3		
12	g.6655824_6655825ins	E 1	6,655,824	5′ UTR	—	33 bp	3 HTZ and 1 HOM				2.7		
12	rs866187118	E 1	6,655,765	5′ UTR	G	T	1 G/T				11.7	0	
12	rs544472399	E 1	6,655,666	5′ UTR	A	G	3 A/G				7.8	0.06	
12	rs12288387	E 1	6,655,653	5′ UTR	T	C	6 T/C and 5 C/C				9.4	0	
12	rs117253802	I 1	6,655,505		C	T	4 C/T				7.5	0.24	
12	rs114655313	I 1	6,655,412		G	A	1 G/A				18.1	0.06	
12	rs16916484	I 1	6,641,975		C	T	3 C/T				3.0	0.14	
12	rs16916480	I 1	6,641,874		G	A	3 G/A				0.7	0.15	
100	rs376287018	E 2	6,641,514	Insertion	—	CTG	19 –/CTG and 2 CTG/CTG	c.99_100insCTG	p.L33dup		11.8	0	0.11
**100**	**rs117368891**	**E 2**	**6,640,369**	**Missense**	**C**	**A**	**3 C/A**	**c.1245C>A**	**p.S415R**	**0.998—P.D.**	**13.4**	**3.00E‐03**	**0.01**
12	rs997263	E 3	6,634,202	Synonymous	C	T	5 C/T and 2 T/T	c.1902C>T	p.H634H		5.8	0.35	0.38
12	rs2659869	I 3	6,634,101		A	T	5 A/T and 2 T/T				0.3	0.39	0.39
12	rs11040938	I 5	6,633,382		T	C	5 T/C and 2 C/C				12.3	0.53	0.50
12	rs138559226	E 7	6,631,745	Synonymous	G	A	1 G/A	c.3546G>A	p.Q1182Q		5.5	0.01	0.01
12	rs2659871	E 8	6,631,387	Synonymous	G	A	5 G/A and 2 A/A	c.3696G>A	p.P1232P		13.5	0.45	0.42
12	rs2659872	E 10	6,630,579	Synonymous	T	C	5 T/C and 2 C/C	c.4215T>C	p.L1405L		7.5	0.53	0.52
12	rs11040937	I 13	6,627,706		G	A	5 G/A and 2 A/A				10.3	0.32	0.37
12	rs4758443	E 14	6,627,193	Missense	C	T	5 C/T and 2 T/T	c.5846C>T	p.T1949M	0.023—benign	0.0	0.30	0.36
12	rs4442534	I 14	6,626,747		G	A	5 G/A and 2 A/A				0.1	0.32	0.36
12	rs34782445	I 14	6,626,700		G	A	5 G/A and 2 A/A				0.0	0.32	0.36
12	rs11827437	I 15	6,626,391		A	G	5 A/G and 2 G/G				0.1	0.34	0.37
12	rs200103093	I 17	6,625,768		—	C	1 –/C				11.8	0.01	0.01
100	rs7924553	E 19	6,625,353	Missense	G	A	3 G/A	c.6991G>A	p.V2331I	0—benign	8.8	0.05	0.02
12	rs10458926	I 19	6,624,877		A	G	3 A/G				0.4	0.20	0.19
**100**	**rs117140835**	**E 21**	**6,624,291**	**Missense**	**G**	**A**	**5 G/A**	**c.7385G>A**	**p.R2462Q**	**1—P.D.**	**25.6**	**0.01**	**0.02**
**100**	**c.7390G>C (New)**	**E 21**	**6,624,286**	**Missense**	**G**	**C**	**1 G/C**	**c.7390G>C**	**p.A2464P**	**0.998—P.D.**	**26.4**	**0**	**0**
100	rs149685502	E 21	6,624,266	Synonymous	G	T	2 G/T	c.7410G>T	p.V2470V		1.7	0	1.63E‐04
100	rs72911011	E 21	6,623,369	Synonymous	G	A	26 G/A and 7 A/A	c.8307G>A	p.A2769A		13.0	0.13	0.24
**100**	**c.8309G>A**	**E21**	**6,623,367**	**Missense**	**G**	**A**	**1 G/A**	**c.8309G>A**	**p.R2770Q**	**0.999—P.D.**	**25.1**	**0**	**0**
**100**	**rs35599968**	**E 21**	**6,623,196**	**Missense**	**G**	**C**	**14 G/C**	**c.8480G>C**	**p.R2827P**	**0.123—benign**	**19.8**	**0.03**	**0.07**
**100**	**rs146233988**	**E 21**	**6,623,077**	**Missense**	**G**	**A**	**1 G/A**	**c.8599G>A**	**p.A2867T**	**0.001—benign**	**15.4**	**4.00E‐04**	**0.01**
100	rs7122587	E 21	6,622,745	Synonymous	G	A	26 G/A and 7 A/A	c.8931G>A	p.Q2977Q		4.1	0.20	0.27
100	rs768737101^a^	E 19	6,625,356	Missense	C	T	0	c.6988C>T	p.R2330C	1—P.D.	28.0	0	2.49E‐05
100	rs201457110^a^	E 21	6,624,138	Missense	G	A	0	c.7538G>A	p.R2513H	1—P.D.	24.3	4.00E‐04	5.05E‐04

I, intron; E, exon; P.D., probably damaging. Sequence of reference: NG_033858.1.

The six mutations considered deleterious in this study are in bold.

a Mutations identified by Durst et al. (2015).

All coding and untranslated regions of the *DCHS1* gene were then sequenced in a subset of 12 patients in order to have a more comprehensive understanding of the mutational burden of this gene in patients with MVP. Six additional synonymous variants were identified in exons 3, 7, 8, 10, and 21 (Figure 1b, Table 2 and Table S2). In addition, one relatively frequent insertion (rs376287018) in exon 2 and 5 missenses were identified. Missenses include p.S415R (rs117368891) on exon 2 classified as probably damaging, p.T1949M on exon 14 (rs4758443) benign, and p.R2827P (rs35599968) and p.A2867T (rs146233988) on exon 21 that are potentially pathogenic with CADD score greater than 15. Finally, a rare missense variant (p.R2770Q) was identified in one heterozygote patient characterized by a G to A substitution (c.8309G>A) resulting of a glutamine instead of an arginine at position 2,770 of the protein (Figure 1a). This rare variant is also considered pathogenic to the same extent as p.R2513H and p.R2330C (Table 2). Taken together, by sequencing all coding regions of *DCHS1* in 12 patients, we identified four additional in silico‐predicted deleterious variants: p.S415R (rs117368891), p.R2827P (rs35599968), p.A2867T (rs146233988), and p.R2770Q. Amplicons with these four variants (exon 2 part 2–1,218 bp and exon 21 part 1–1,883 bp, see Table S1) were then sequenced in the remaining 88 samples to obtain a more accurate estimate of their frequencies (Table 2 and Figure 1b). The location of identified variants in exons and corresponding protein domains is illustrated in Fig. S1.

Figure 1c summarizes patients carrying at least one of six variants identified and considered deleterious in this study. In total, 24 variant carriers are illustrated including one patient with two variants, suggesting an important contribution of germline variants in *DCHS1* in unrelated patients with mitral regurgitation.

## 4 DISCUSSION

Two loss‐of‐function variants (p.R2513H and p.R2330C) in *DCHS1* were identified as causing nonsyndromic MVP in three families (Durst et al., 2015). Whether these variants are restricted to few families or in individuals from specific geographic region had to be determined. In this study, the two variants were absent in 100 sporadic cases of MVP. However, the present work underscores that other and similarly pathogenic variants in *DCHS1* are frequently observed in patients with MVP. A total of 24 of 100 cases were carriers of at least one in silico‐predicted deleterious missense variant in *DCHS1*. The frequencies of these individual variants are rare in populations of reference, which suggests a clear enrichment among cases. In addition, by performing our full 21‐exon screening in 12 patients, we cannot exclude the possibility that other rare damaging *DCHS1* variants are present in the remaining 88 patients. Variants in this single gene may thus account for a substantial portion of patients with MVP. Overall, this study identified inherited variants likely causing MVP in sporadic cases and further supports the role of *DCHS1* in the disease pathogenesis. The variety of variants also emphasized for a more comprehensive evaluation of this gene to screen individuals at risk.

## CONFLICT OF INTEREST

The authors declare no conflict of interest.
