# Supplementary figures and images for: Deleterious variants in DCHS1 are prevalent in sporadic cases of mitral valve prolapse
Source: Mol Genet Genomic Med. 2017 Dec 10;6(1):114–20. doi: 10.1002/mgg3.347 (PMC5823682; doi:10.1002/mgg3.347)

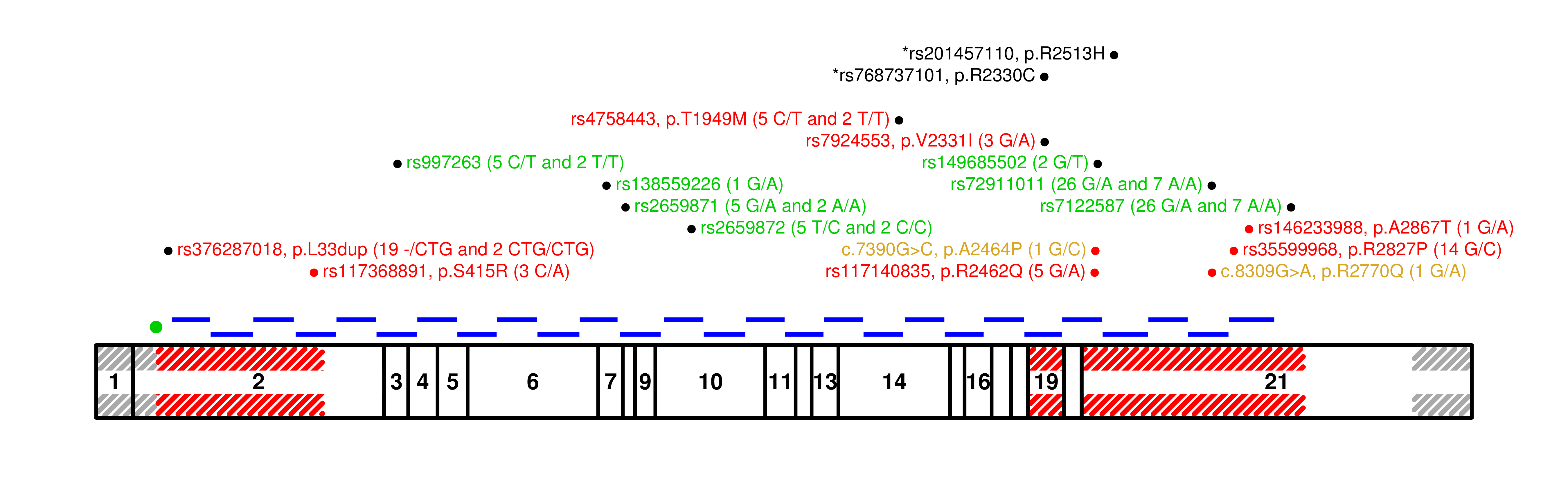

Supplement: Supplementary file 1 [file MGG3-6-114-s001.png]
