# Supplementary material for: Deleterious variants in DCHS1 are prevalent in sporadic cases of mitral valve prolapse
Source: Mol Genet Genomic Med. 2017 Dec 10;6(1):114–20. doi: 10.1002/mgg3.347 (PMC5823682; doi:10.1002/mgg3.347)
Supplement: Supplementary file 2 [file MGG3-6-114-s002.docx]

**Supplementary Table S1**. Primers to sequence the *DCHS1* gene

|  | **Forward 5’ 🡪 3’** | **Reverse 5’ 🡪 3’** | **Amplicon (bp)** | **PCR reagent*** | **PCR cycling**** | **Annealing T ⁰C** |
| --- | --- | --- | --- | --- | --- | --- |
| Exon 1 | **GATGGAAGAGGAGGAGGTGAGC** | **GACACGCACATGCACACACAG** | 947 | B | 5 | Slowdown |
| Exon 2 (part 1) | GATGGAAAGCTCCAGGAGAGAGGAAA | **ACGCCCAGCATCTGCATCA** | 1060 | A | 1 | 61 |
| Exon 2 (part 2) | GAGCCTGGACTTGCAGATTGATGA | **GGAGGTGAGCCTGAGTCTGTG** | 1218 | A | 1 | 61 |
| Exon 2 (part 3) | CAATGTGTCCCTGGAAGGTGGAGA | **CCTGCCAAGACCATCTGCCTC** | 815 | A | 1 | 61 |
| Exon 2  rs376287018  rs117368891 | **GATGGAAAGCTCCAGGAGAGAGGAAA** | **GGAGGTGAGCCTGAGTCTGTG** | 1890 | A | 1 | 61 |
| Exon 3-4-5 | **GTCCGCCATCCAGAGACTTAGC** | **GCCTGCACTGTGCCTAAGGA** | 1160 | A | 1 | 60 |
| Exon 6 | **AAAGGCATGGGTGTGGGCA** | **TCCTGTCTGAATGTTCACCAGGCT** | 1204 | A | 2 | Touchdown |
| Exon 7-8-9 | **CCCAGCCTGGTGAACATTCAGAC** | AGTAGCCTGACTGCTAGTGGAGC | 1034 | A | 3 | Touchdown |
| Exon 10 (part 1) | **GCTCCACTAGCAGTCAGGCTACT** | GCCTGGACCGAGAGACCA | 700 | A + 5% DMSO | 4 | Touchdown |
| Exon 10 (part 2) | **CAAGTGCAGGACGAGAATGAGCAT** | CGTCGTTGACGTCAGCGACA | 748 | A | 1 | 62 |
| Exon 11 | **GCGGCCTGGACCGAGAG** | CTTGGGGTCCTGTCAACATGTAC | 870 | A | 1 | 58 |
| Exon 12 | GTGTCGCTGACGTCAACGAC | **GTTGACCTTGCACCCTGCC** | 517 | A | 1 | 58 |
| Exon 13 | **GAAGTGGCAGGGCTGGGA** | CAAGAAGGAGCAAGAACCAGGCAAG | 555 | A | 1 | 61 |
| Exon 14-15 | **GGGTTCCCAGACCACACACA** | **CCATCAAAGGCTCCTCTGATGCAA** | 1389 | A | 2 | Touchdown |
| Exon 16-17-18 | **TTGCATCAGAGGAGCCTTTGATGG** | **GGGCTTAGCACATACCACAGCA** | 1096 | A | 1 | 61 |
| Exon 19  rs768737101 | **TGCTGATGGCCACAGACAGA** | AGGTGGATCCATGGGTGTCAA | 551 | A | 1 | 58 |
| Exon 19-20 | **TCTCCACCCTGCAGCTCAAG** | ACTCAGGGAATGGCCTATCTGCT | 1058 | A | 1 | 60 |
| Exon 21  rs201457110 | **AGCTGTGAATCCTTGAGGCCA** | GGTCAGCTGCAGCCACTG | 602 | A | 1 | 58 |
| Exon 21 (part 1) | **AGCAGATAGGCCATTCCCTGAGT** | **CAGCTCCTGGCCCACCATAG** | 1883 | A | 2 | Touchdown |
|  |  | **CATCCTCATCTGTGGCCTGC** |  |  |  |  |
| Exon 21 (part 2) | **CTTGCACTGGCAGCCCTG** | **CCTGAGCTCCCAGTGCCAT** | 1610 | A | 2 | Touchdown |
|  |  | **TCTGTGGTCCTAGTACTCTGAGGG** |  |  |  |  |

**Bold=** sequencing primers

All PCR reactions were carried out on a GeneAmp^®^ PCR system 9700 (Applied Biosystems) or a Veriti thermal cycler (Applied Biosystems) in a final volume of 25 μl containing:

***PCR reagent**

**A:** 100 ng DNA, 1X PCR buffer Qiagen, 0.2µM of each primer, 160 µM of each dNTP, 1X Q-solution Qiagen, 1 Unit HotStarTaq Qiagen.

**B**: 100 ng DNA, 1X PCR buffer Qiagen, 0.4µM of each primer, 160 µM of each dNTP, 1X Q-solution Qiagen, 160 µM of 7-deaza-dGTP, 1 Unit HotStarTaq Qiagen.

****PCR cycling**

**1:** 15 minutes at 95^o^C, 35 PCR amplification cycles (15 seconds at 94^o^C, 30 seconds at annealing temperature, and 60 to 120 seconds at 72^o^C), and 5 minutes at 72^o^C.

**2:** (Touchdown) 15 minutes at 95^o^C, 15 PCR amplification cycles (15 seconds at 94^o^C, 30 seconds starting at 67 ^o^C, and -0.5 ^o^C/cycle, and 2 min at 72^o^C), 35 PCR amplification cycles (15 seconds at 94^o^C, 30 seconds at 60 ^o^C and 2 minutes at 72^o^C) and 5 minutes at 72^o^C.

**3:** (Touchdown) 15 minutes at 95^o^C, 11 PCR amplification cycles (15 seconds at 94^o^C, 30 seconds starting at 67 ^o^C, and -0.5 ^o^C/cycle, and 2 min at 72^o^C), 24 PCR amplification cycles (15 seconds at 94^o^C, 30 seconds at 62 ^o^C and 2 minutes at 72^o^C) and 5 minutes at 72^o^C.

**4:** (Touchdown) 15 minutes at 95^o^C, 15 PCR amplification cycles (15 seconds at 94^o^C, 30 seconds starting at 67 ^o^C, and -0.5 ^o^C/cycle, and 2 min at 72^o^C), 20 PCR amplification cycles (15 seconds at 94^o^C, 30 seconds at 58 ^o^C and 2 minutes at 72^o^C) and 5 minutes at 72^o^C.

**5:** (Slowdown) 15 minutes at 95^o^C, 41 PCR amplification cycles (30 seconds at 95^o^C, 30 seconds starting at 78 ^o^C, and -0.3 ^o^C/cycle, and 1 min at 72^o^C), 15 PCR amplification cycles (30 seconds at 94^o^C, 30 seconds at 58 ^o^C and 1 minutes at 72^o^C) and 5 minutes at 72^o^C.
